# Supplementary material for: Understanding the spatiotemporal pattern of grazing cattle movement
Source: Sci Rep. 2016 Aug 24;6:31967. doi: 10.1038/srep31967 (PMC4995401; doi:10.1038/srep31967)
Supplement: Supplementary Information [file srep31967-s1.pdf]

Supporting information

Understanding the spatiotemporal pattern of grazing cattle movement
By Kun Zhao, Raja Jurdak

Table S1. The AIC weights for the population trip length distribution

Δt	Powerlaw	Truncated powerlaw	Exponential	Hybrid exponential
60	0.00	0.00	0.00	**1.00**
120	0.00	0.00	0.00	**1.00**
180	0.00	0.00	0.00	**1.00**

Table S2. The AIC weights for the population waiting time distribution

Δt	Powerlaw	Truncated powerlaw	Exponential
60	0.00	**1.00**	0.00
120	0.00	**1.00**	0.00
180	**0.52**	0.48	0.00

Table S3. The AIC weights for the individual trip length distribution

Cow ID	Powerlaw	Truncated powerlaw	Exponential	Hybrid exponential
0	0.00	0.40	0.00	**0.60**
1	0.00	0.01	0.00	**0.99**
2	0.00	0.06	0.00	**0.94**
4	0.00	0.35	0.00	**0.65**
5	0.00	0.08	0.00	**0.92**
6	0.00	0.23	0.00	**0.77**
7	0.00	0.05	0.00	**0.95**
8	0.00	0.14	0.00	**0.86**
9	0.00	0.01	0.00	**0.99**
10	0.00	0.11	0.00	**0.89**
12	0.00	0.47	0.00	**0.53**
13	0.00	0.14	0.00	**0.86**
14	0.00	0.01	0.00	**0.99**
16	0.00	0.40	0.00	**0.60**
17	0.00	0.22	0.00	**0.78**
18	0.00	0.02	0.00	**0.98**
19	0.00	0.05	0.00	**0.95**
20	0.00	0.03	0.00	**0.97**
21	0.00	0.01	0.00	**0.99**
22	0.00	**0.56**	0.00	0.44
23	0.00	0.33	0.00	**0.67**
24	0.00	0.22	0.00	**0.78**
25	0.00	0.00	0.00	**1.00**
26	0.00	0.02	0.00	**0.98**
27	0.00	0.01	0.00	**0.99**
29	0.00	0.17	0.00	**0.83**
30	0.00	0.45	0.00	**0.55**
32	0.00	0.27	0.00	**0.73**
33	0.00	0.22	0.00	**0.78**

Table S4. The AIC weights for the individual trip time distribution

Cow ID	Powerlaw	Truncated powerlaw	Exponential	Hybrid exponential
0	0.00	0.00	0.00	**1.00**
1	0.00	0.00	0.00	**1.00**
2	0.00	0.00	0.00	**1.00**
4	0.00	0.00	0.00	**1.00**
5	0.00	0.00	0.00	**1.00**
6	0.00	0.00	0.00	**1.00**
7	0.00	0.00	0.00	**1.00**
8	0.00	0.00	0.00	**1.00**
9	0.00	0.00	0.00	**1.00**
10	0.00	0.00	0.00	**1.00**
12	0.00	0.00	0.00	**1.00**
13	0.00	0.00	0.00	**1.00**
14	0.00	0.00	0.00	**1.00**
16	0.00	0.00	0.00	**1.00**
17	0.00	0.00	0.00	**1.00**
18	0.00	0.00	0.00	**1.00**
19	0.00	0.00	0.00	**1.00**
20	0.00	0.00	0.00	**1.00**
21	0.00	0.00	0.00	**1.00**
22	0.00	0.00	0.00	**1.00**
23	0.00	0.00	0.00	**1.00**
24	0.00	0.00	0.00	**1.00**
25	0.00	0.00	0.00	**1.00**
26	0.00	0.00	0.00	**1.00**
27	0.00	0.00	0.00	**1.00**
29	0.00	0.00	0.00	**1.00**
30	0.00	0.00	0.00	**1.00**
32	0.00	0.00	0.00	**1.00**
33	0.00	0.00	0.00	**1.00**

Table S5. The AIC weights for the individual waiting time distribution

Cow ID	Powerlaw	Truncated powerlaw	Exponential
0	**0.66**	0.34	0.00
1	**0.66**	0.34	0.00
2	**0.71**	0.29	0.00
4	**0.72**	0.28	0.00
5	**0.68**	0.32	0.00
6	**0.66**	0.34	0.00
7	0.49	**0.51**	0.00
8	**0.73**	0.27	0.00
9	**0.73**	0.27	0.00
10	**0.66**	0.34	0.00
12	**0.62**	0.38	0.00
13	**0.58**	0.42	0.00
14	**0.66**	0.34	0.00
16	**0.73**	0.27	0.00
17	**0.73**	0.27	0.00
18	**0.72**	0.28	0.00
19	0.48	**0.52**	0.00
20	0.48	**0.52**	0.00
21	**0.71**	0.29	0.00
22	0.36	**0.64**	0.00
23	0.47	**0.53**	0.00
24	**0.61**	0.39	0.00
25	**0.70**	0.30	0.00
26	**0.70**	0.30	0.00
27	**0.71**	0.29	0.00
29	**0.68**	0.32	0.00
30	0.49	**0.51**	0.00
32	**0.62**	0.38	0.00
33	**0.62**	0.38	0.00
